# Single-Molecule Sequencing of the *C9orf72* Repeat Expansion in Patient iPSCs

**DOI:** 10.21769/BioProtoc.5060

**Published:** 2024-09-05

**Authors:** Yu-Chih Tsai, Katherine A. Brown, Mylinh T. Bernardi, John Harting, Claire D. Clelland

**Affiliations:** 1 Pacific Biosciences, Menlo Park, CA, USA; 2 Weill Institute for Neurosciences, University of California San Francisco, San Francisco, CA, USA; 3 Memory & Aging Center, Department of Neurology, University of California San Francisco, San Francisco, CA, USA; 4 Gladstone Institutes, San Francisco, CA, USA; 5 Innovative Genomics Institute, Berkeley, CA, USA

**Keywords:** *C9orf72*, Single-molecule sequencing, No amplification, Repeat expansion, iPSCs

## Abstract

A hexanucleotide GGGGCC repeat expansion in the *C9orf72* gene is the most frequent genetic cause of amyotrophic lateral sclerosis (ALS) and frontal temporal dementia (FTD). *C9orf72* repeat expansions are currently identified with long-range PCR or Southern blot for clinical and research purposes, but these methods lack accuracy and sensitivity. The GC-rich and repetitive content of the region cannot be amplified by PCR, which leads traditional sequencing approaches to fail. We turned instead to PacBio single-molecule sequencing to detect and size the *C9orf72* repeat expansion without amplification. We isolated high molecular weight genomic DNA from patient-derived iPSCs of varying repeat lengths and then excised the region containing the *C9orf72* repeat expansion from naked DNA with a CRISPR/Cas9 system. We added adapters to the cut ends, capturing the target region for sequencing on PacBio’s Sequel, Sequel II, or Sequel IIe. This approach enriches the *C9orf72* repeat region without amplification and allows the repeat expansion to be consistently and accurately sized, even for repeats in the thousands.

Key features

• This protocol is adapted from PacBio’s previous “no-amp targeted sequencing utilizing the CRISPR-Cas9 system.”

• Optimized for sizing *C9orf72* repeat expansions in patient-derived iPSCs and applicable to DNA from any cell type, blood, or tissue.

• Requires high molecular weight naked DNA.

• Compatible with Sequel I and II but not Revio.

## Graphical overview


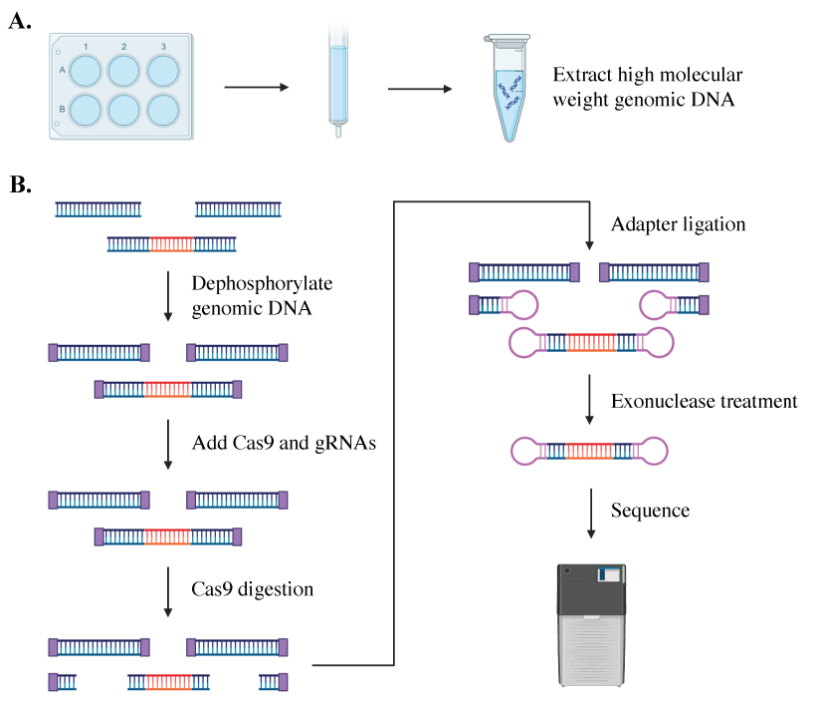


## Background

The most frequent genetic cause of amyotrophic lateral sclerosis (ALS) and frontotemporal dementia (FTD) is an intronic repeat expansion in the *C9orf72* gene [1–3]. While non-diseased alleles typically have 10 or fewer GGGGCC repeats, expanded alleles can have thousands [1,2,4,5]. Accurately measuring the *C9orf72* repeat expansion length applies to both clinical diagnosis and answering fundamental questions about the function of *C9orf72* in health and disease. Traditional short-read sequencing methods fail to size the *C9orf72* repeat region because amplification fails across the GC-rich region of the first intron of *C9orf72*, and short-read sequencing does not provide unique sequences adjacent to GGGGCC repeats to permit their alignment [5]. Instead, long-range PCR [6] and Southern blot [7] are used as clinical diagnostic and research tools to identify *C9orf72* repeat expansions, but they are limited. Long-range PCR fails for repeats greater than 145 and, while Southern blot can identify long repeats, it requires a large amount of input DNA [5].

Single-molecule sequencing is an accurate, reliable, and sensitive method to size repeat lengths of varied sizes, showing success in sequencing the *C9orf72* repeat expansion in both plasmid and tissue [5,8–10]. This amplification-free process employs a CRISPR/Cas9 system to excise and sequence the region containing the *C9orf72* expansion in high-quality DNA from patient iPSCs (see Graphical overview). The excised genomic fragment is then sequenced from end to end, providing phased sequencing of the targeted region without the need for bioinformatic imputation of its sequence or structure. In addition to identifying and quantifying repeat expansions of various lengths, the sequencing data provides insights into the gene structure. Single-molecule sequencing can identify mosaicism within samples and help determine the stability of repeat lengths over time [5,8,10]. It can be used to assess cell line clonality, editing outcomes, and differences between each allele of a gene in a single individual [5,10]. This method provides additional information on the methylation of each allele within an individual or across a population [10].

Despite the technological advances afforded by single-molecule sequencing, there are a few limitations to employing this method in research and clinical settings. The first is cost. Single-molecule sequencing is expensive. The ability to multiplex samples, as we describe here, reduces the cost of this protocol. The second limitation is the variability in read depth with longer repeat expansions. As the size of repeat expansion increases, the number of reads on the expanded allele tends to decrease [5]. Long expansions can still be sized, albeit with reduced read depth. Over time, as technology improves and the number of reads for each run increases, this bias is likely to decrease.

We have optimized the protocol presented here for genomic DNA extracted from human induced pluripotent stem cell (iPSC) lines derived from C9-ALS/FTD patient samples. This protocol can also be applied to other sources of DNA such as any cultured cell type as well as donor blood and fresh or frozen tissue. This method can also be used to target other repeat expansions such as TCF4 and DPMK, for which this approach was initially reported [11,12], or other genomic regions of interest by targeting the Cas9/gRNA excision to the target region. This protocol is compatible with Sequel I and Sequel II. PacBio’s PureTarget^TM^ Repeat Expansion Panel [13], which is an improved version of the NoAmp protocol, can be used additionally with Revio and quantifies 20 different repeat expansions for targeted single-molecule sequencing simultaneously and in the same individual.

## Materials and reagents

**Biological materials**

1. 5–25 μg of DNA from induced pluripotent stem cells (iPSCs) with *C9orf72* repeat expansions (also compatible with DNA from blood or tissue)

**Reagents**

1. DPBS (Gibco, catalog number: 14190235)
2. ReLeSR (STEMCELL Technologies, catalog number: 05872)
3. Genomic DNA buffer set (QIAGEN, catalog number: 19060)
4. Proteinase K (Qiagen, catalog number: 19131)
5. Qubit^TM^ 1× dsDNA HS Assay kit (Thermo Fisher Scientific, catalog number: Q33230)
6. Agarose (Fisher Scientific, catalog number: BP164-500)
7. 50× TAE buffer (Thermo Fisher Scientific, catalog number: B49)
8. SYBR^TM^ Safe DNA gel stain (Thermo Fisher Scientific, catalog number: S33102)
9. 6× gel loading dye (New England BioLabs, catalog number: B7024S)
10. Nuclease-free water, not DEPC treated (Ambion, catalog number: AM9937)
11. Ethanol, molecular biology grade
12. Isopropyl alcohol, molecular biology grade
13. Custom single-guide RNA (sgRNA 1) TTGGTATTTAGAAAGGTGGT (Synthego)
14. Custom single-guide RNA (sgRNA 2) GGAAGAAAGAATTGCAATTA (Synthego)
15. 1× TE buffer (included with sgRNA orders from Synthego)
16. Custom barcoded adapter oligos (IDT)
*Note: See Table 1 for a list of barcoded adapter oligos.*
  **Table 1.** Barcoded adapters

Barcoded adapter	Sequence
Barcoded adapter 1	/5Phos/CGCACTCTGATATGTGATCTCTCTCTTTTCCTCCTCCTCCGTTGTTGTTGTTGAGAGAGATCACATATCAGAGTGCG
Barcoded adapter 2	/5Phos/CTCACAGTCTGTGTGTATCTCTCTCTTTTCCTCCTCCTCCGTTGTTGTTGTTGAGAGAGATACACACAGACTGTGAG
Barcoded adapter 3	/5Phos/CGCAGCGCTCGACTGTATCTCTCTCTTTTCCTCCTCCTCCGTTGTTGTTGTTGAGAGAGATACAGTCGAGCGCTGCG
Barcoded adapter 4	/5Phos/TCTGTCTCGCGTGTGTATCTCTCTCTTTTCCTCCTCCTCCGTTGTTGTTGTTGAGAGAGATACACACGCGAGACAGA
Barcoded adapter 5	/5Phos/CTCTGAGATAGCGCGTATCTCTCTCTTTTCCTCCTCCTCCGTTGTTGTTGTTGAGAGAGATACGCGCTATCTCAGAG
Barcoded adapter 6	/5Phos/ACACGCGATCTAGTGTATCTCTCTCTTTTCCTCCTCCTCCGTTGTTGTTGTTGAGAGAGATACACTAGATCGCGTGT
Barcoded adapter 7	/5Phos/ACGCGCGCGTAGTGAGATCTCTCTCTTTTCCTCCTCCTCCGTTGTTGTTGTTGAGAGAGATCTCACTACGCGCGCGT
Barcoded adapter 8	/5Phos/ACACACGTGTCATGCGATCTCTCTCTTTTCCTCCTCCTCCGTTGTTGTTGTTGAGAGAGATCGCATGACACGTGTGT
Barcoded adapter 9	/5Phos/ATACTATCTCTCTATGATCTCTCTCTTTTCCTCCTCCTCCGTTGTTGTTGTTGAGAGAGATCATAGAGAGATAGTAT
Barcoded adapter 10	/5Phos/CACAGTGAGCACGTGAATCTCTCTCTTTTCCTCCTCCTCCGTTGTTGTTGTTGAGAGAGATTCACGTGCTCACTGTG
17. Shrimp alkaline phosphatase (rSAP) (New England BioLabs, catalog number: M0371S or M0371L)
18. CutSmart^®^ buffer 10× (New England BioLabs, catalog number: B7204S)
19. Exonuclease III (New England BioLabs, catalog number: M0206S or M0206L)
20. Cas9 nuclease, *S. pyogenes* (New England BioLabs, catalog number: M0386T or M0386M)
21. NEBuffer^TM^ 3.1, 10× (New England BioLabs, catalog number: B7203S)
*Note: Included with Cas9 Nuclease*, S. pyogenes *from New England BioLabs.*
22. T4 DNA ligase reaction buffer, 10× (New England BioLabs, catalog number: B0202S)
23. T4 DNA ligase, HC (Thermo Fisher Scientific, catalog number: EL0013)
24. SOLu-Trypsin (Sigma-Aldrich, catalog number: EMS0004)
25. 1 kb DNA ladder (carrier DNA) (New England BioLabs, catalog number: N3232S or N3232L)
26. Recombinant ribonuclease inhibitor (Takara Bio, catalog number: 2313A or 2313B)
27. Pacific Biosciences^®^ Binding Kits
  For Sequel: Sequel Binding and Internal Control kit 3.0 (PacBio, catalog number: 101-626-600)
  For Sequel II and IIe: Sequel II Binding kit 2.0 and Internal Control kit 1.0 (PacBio, catalog number: 101-842-900)
28. Pacific Biosciences^®^ Sequencing Kits
  For Sequel: Sequel Sequencing kit 3.0 (PacBio, catalog number: 101-597-900)
*Note: Four reactions per kit.*
  For Sequel II and IIe: Sequel II Sequencing kit 2.0 (PacBio, catalog number: 101-820-200)
*Note: Four reactions per kit.*
29. Pacific Biosciences^®^ SMRT^®^ cells
30. For Sequel: SMRT^®^ Cell 1M v3 tray or SMRT^®^ Cell 1M v3 LR tray (PacBio, catalog number: 101-531-000 or 101-531-001)
31. For Sequel II and IIe: SMRT Cell 8M tray (PacBio, catalog number: 101-389-001)
32. AMPure^®^ PB beads (PacBio, catalog number: 100-265-900)
33. Elution buffer (PacBio, catalog number: 101-633-500)
34. No-Amp Accessory kit (PacBio, catalog number: 101-788-900)
  Sequencing primer v4
  10× primer buffer v2
  10× annealing buffer
35. SMRTbell^®^ Enzyme Clean-up kit (PacBio, catalog number: 101-746-400)
36. Sample plate (PacBio, catalog number: 000-448-888)

**Solutions**

1. 80% ethanol (see Recipes)

**Recipes**

1. **80% ethanol**
  **Table:** 

Reagent	Final concentration	Amount
Ethanol (absolute)	80%	40 mL
Nuclease-free H_2_O	n/a	10 mL
Total	n/a	50 mL

**Laboratory supplies**

1. 15 mL conical tubes (Falcon, catalog number: 352096)
2. 50 mL conical tube (Falcon, catalog number: 1443222)
3. QIAGEN^®^ genomic tip 100/G (QIAGEN, catalog number: 10243)
4. Qubit^TM^ assay tubes (Thermo Fisher Scientific, catalog number: Q32856)
5. 1.5 mL DNA LoBind tubes (Eppendorf, catalog number: 0030108051)
6. 0.2 mL PCR thermal cycling tubes (Genesee Scientific, catalog number: 27-125)

## Equipment

1. Molecular biology pipettes, standard set
2. Avanti^TM^ J-15R, IVD, refrigerated benchtop centrifuge (or equivalent) (Beckman Coulter, catalog number: B99517)
3. Platform shaker
4. Qubit^TM^ quantitation platform (Thermo Fisher Scientific, catalog number: Q33238)
5. Gel imaging system
6. Benchtop cooler
7. Microcentrifuge
8. Mini centrifuge
9. Vortex mixer
10. DynaMag^TM^-2 magnet (magnetic rack) (Thermo Fisher Scientific, catalog number: 12321D)
11. PCR thermal cycler
12. ThermoMixer C with heated lid (or equivalent) (Eppendorf, catalog number: 5382000023)
13. Sequel, Sequel II, or Sequel IIe Systems (Pacific Biosciences)

## Software and datasets

1. SMRTLink (v12.0, 2023)
2. ccs (v7.0.0, https://github.com/PacificBiosciences/ccs)
3. lima (v2.7.1, 2023)
4. pbmm2 (v1.10.0, 2023)
5. primrose (v1.2.0, 2022, https://github.com/mattoslmp/primrose)
6. pb-CpG-tools (v2.3.2, 2023, https://github.com/PacificBiosciences/pb-CpG-tools)

## Procedure

- **Genomic DNA extraction**
  1. Grow iPSCs in 2 wells of a 6-well plate to 80% confluency [14].
  2. Aspirate the media and wash the cells once with 1 mL of DPBS.
  3. Add 1 mL of ReLeSR to each well and incubate in the hood for 45 s.
  4. Aspirate the ReLeSR and incubate at 37 °C for 3 min.
  5. Resuspend the wells in 1 mL of DPBS each and transfer to a 15 mL conical tube.
  6. Centrifuge at 300× *g* for 3 min.
  7. Remove the supernatant.
  8. Cell pellets may be immediately used for DNA extraction or can be stored at -20 °C.
  9. To extract DNA from the cell pellet, follow the QIAGEN^®^ Genomic DNA Handbook [15] for 100/G tips with the following modifications:
    - After adding 95 μL of Proteinase K, incubate at 55 °C for 90 min.
    - After precipitating with 3.5 mL of isopropyl alcohol, invert the tube four times and immediately centrifuge at 4,000× *g* for 20 min at 4 °C. Then, remove the supernatant.
    - After the ethanol wash, centrifuge at 4,000× *g* for 20 min at 4 °C and then remove the supernatant.
    - After the pellet air dries for 10 min, resuspend DNA in 50 μL of Buffer EB. At room temperature, dissolve the DNA in the elution buffer overnight on a shaker at 300 rpm.
  10. Aliquot 3 μL of DNA for quality control and store the rest at -20 °C.
- **Evaluate DNA quality**
  1. Measure the concentration of your genomic DNA using the Qubit^TM^ 1× dsDNA HS Assay kit and associated protocol [16].
*Note: Prior to measuring the concentrations, dilute genomic DNA 1:10.*
  2. It is recommended to evaluate sample quality with the Agilent FEMTO Pulse System, the Bio-Rad CHEF Mapper XA Pulsed Field Electrophoresis System, or the Sage Science Pippin Pulse Electrophoresis Power Supply. Genomic DNA samples should have fragment sizes ≥ 50 kb. Significant on-target reads can still be achieved with fragment sizes <50 kb by increasing the amount of input DNA, but better results may be achieved by collecting a fresh sample to achieve fragment sizes ≥ 50 kb.
*Note: We skip this step. Instead, we evaluate DNA quality with a 1% agarose gel as described in steps B4–7 (Figure 1).*
  3. Prepare a 1% agarose gel with SYBR^TM^ Safe DNA gel stain [17].
  4. Add 2 μL of genomic DNA, 7 μL of water, and 1 μL of 6× gel loading dye to a PCR tube.
  5. Pipette-mix gently and load the diluted DNA into the 1% gel.
  6. Run the gel at 100 V for 1 h.
  7. Image the gel and check for any smearing in your samples. Samples with a single band above 10 kb should be included. Samples that have smearing down the gel, even if they have the >10 kb band, indicate DNA shearing and should be excluded from the protocol. In the case of shearing, fresh genomic DNA samples should be collected.
    **Figure 1.**
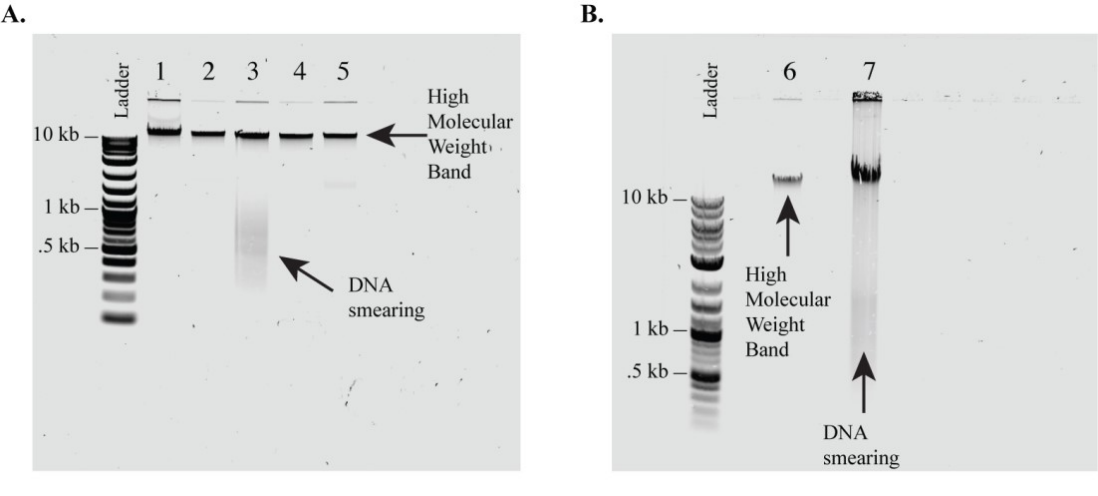
1% Agarose gel evaluation of genomic DNA quality. As a quick quality check, we run isolated DNA on a 1% agarose gel prior to initiating CRISPR isolation of the target region. We include only DNA that has high molecular weight (i.e., has no smearing). **(A, B)** Examples of genomic DNA samples extracted from *C9orf72* patient iPSC lines and run on a 1% agarose gel to evaluate sample quality. (A) Samples 1, 2, 4, and 5 (A) and 6 (B) show no smearing and would be acceptable for use in the protocol. Samples 3 (A) and 7 (B) show significant smearing and therefore we recommend being excluded from this protocol. This is a quick quality check. Sample quality can be confirmed with higher accuracy by the Agilent FEMTO Pulse System, the Bio-Rad CHEF Mapper XA Pulsed Field Electrophoresis System, or the Sage Science Pippin Pulse Electrophoresis Power Supply.
- **Prepare reagents**
  Prior to starting the library preparation, prepare the PacBio barcoded adapters for multiplexing and the carrier DNA.
  1. To prepare the PacBio barcoded adapters for multiplexing, resuspend the barcoded adapter oligos in nuclease-free water to 100 μM.
  2. Add the following reagents, in order, to a PCR tube to make 20 μM stocks of barcoded adapters:
    100 μM barcoded adapter oligo 10 μL
    10× annealing buffer 5 μL
    Nuclease-free water 35 μL
  3. Place the 20 μM stocks in a thermal cycler and run the following protocol:
    95 °C for 5 min
    Decrease to 25 °C, ramping down at the maximum cooling rate
    4 °C hold
  4. Store stocks at -20 °C.
  5. To prepare the carrier DNA (1 kb DNA ladder), add 5 μL of ladder to 45 μL of elution buffer in a PCR tube to dilute the carrier DNA to a concentration of 50 ng/μL.
  6. Store at -20 °C.
- **Dephosphorylate the genomic DNA**
  1. In a PCR tube, dilute each genomic DNA sample to 5 μg with nuclease-free water for a total volume of 68 μL.
*Note: When multiplexing five samples, 5 μg per sample is the optimal input. If using a different number of samples (1–10) for this protocol, the minimum total DNA input is 5 μg and the maximum total DNA input is 25 μg. Each multiplexed sample should have equimolar input amounts.*
  2. Add the following reagents, in order, to a LoBind microcentrifuge tube to make a master mix. Add 1 volume of each reagent per sample with 10% overage:
    NEBuffer 8 μL
*rSAP* 4 μL
  3. Add 12 μL of dephosphorylation master mix to each sample for a total volume of 80 μL.
  4. Invert the tubes 20 times to mix.
*Note: Do not vortex or flick the tube to avoid shearing the DNA.*
  5. Briefly spin down the tubes in a mini centrifuge.
  6. Place in the thermal cycler and run the following protocol:
    37 °C for 1 h
    65 °C for 10 min
    4 °C hold
- **Prepare the single-guide RNAs (sgRNAs)**
  1. Spin down lyophilized sgRNAs.
  2. Resuspend sgRNAs in TE buffer to 50 μM.
  3. Aliquot sgRNAs and store at -80 °C to avoid freeze-thawing.
  4. Dilute a 50 μM aliquot of each sgRNA to 5 μM.
  5. In a PCR tube, add 4 μL of sgRNA1 and 4 μL of sgRNA2 per sample to make a master mix of diluted sgRNAs. You will need 4 μL of each gRNA per sample with 10% overage.
- **Prepare the gRNA/Cas9 complex**
  1. Add the following reagents, in order, to a LoBind microcentrifuge tube to make a master mix. Add 1 volume of each reagent per sample with 10% overage:
    Nuclease-free water 7 μL
    NEBuffer 3.1 2 μL
    Cas9 nuclease 2 μL
    Diluted sgRNAs (from section E) 8 μL
  2. Pipette-mix the reaction.
  3. Briefly spin down the tube in a mini centrifuge.
  4. Transfer the master mix to a PCR tube and place it in a thermal cycler.
  5. Incubate at 37 °C for 10 min.
  6. Place the gRNA/Cas9 complex on ice.
- **Cas9 digestion**
  1. To each tube with dephosphorylated DNA, add the following reagents, in order, for a total volume of 100 μL:
    Recombinant ribonuclease inhibitor 1 μL
    gRNA/Cas9 complex (from section F) 19 μL
  2. Invert the tubes 20 times to mix.
*Note: Do not vortex or flick the tube to avoid shearing the DNA.*
  3. Briefly spin down the tubes in a mini centrifuge.
  4. Incubate at 37 °C for 1 h.
  5. Briefly spin down the tubes in a mini centrifuge and then place on ice.
- **Purify Cas9-digested DNA**
  1. Transfer the samples to new LoBind microcentrifuge tubes.
  2. Add 400 μL of elution buffer to each sample for a total volume of 500 μL.
  3. Mix the AMPure PB beads until a homogeneous solution forms.
  4. Add 0.45× volume of AMPure PB beads to each tube for a total volume of 725 μL.
*Note: The AMPure PB beads will be viscous; pipette slowly to ensure precise volume.*
  5. Invert the tubes 20 times to mix.
*Note: Do not vortex or flick the tube to avoid shearing the DNA.*
  6. Briefly spin down the tubes in a mini centrifuge.
  7. Incubate for 15 min at room temperature.
  8. While the reaction is incubating, prepare a solution of 80% ethanol (see Recipes) in a 50 mL tube. Store in a tightly capped polypropylene tube for a maximum of three days.
  9. After 15 min, briefly spin down the tubes in a mini centrifuge.
  10. Place the tubes in the magnetic tube rack.
  11. Allow the beads to separate from the liquid and collect on the side of the tube. Wait until the solution appears clear (at least 5 min).
  12. While the tubes are on the magnetic rack, slowly remove the supernatant without disturbing the bead pellet and transfer to new LoBind microcentrifuge tubes.
*Note: Save supernatant to repeat purification steps in case insufficient DNA is recovered at the end of the purification protocol.*
  13. Wash the beads with 1 mL of 80% ethanol by slowly adding it to the side opposite the beads.
*Note: Leave the tubes on the magnetic rack during the ethanol washes. Do not invert the tubes.*
  14. Wait 30 s, then slowly pipette up the ethanol and discard.
  15. Repeat the ethanol wash two more times.
  16. Remove the tubes from the magnetic rack and briefly spin them down in a mini centrifuge.
*Note: Both the beads and ethanol should be at the bottom of the tubes.*
  17. Return the tubes to the magnetic rack.
  18. With a P20 pipette, slowly pipette up any remaining ethanol and discard.
  19. Repeat steps H16–18 until no ethanol droplets remain.
  20. Remove the tubes from the magnetic rack and add 31 μL of elution buffer to each tube.
  21. Invert the tube 10 times to mix.
  22. If the beads are stuck on the tube wall, leave the tube on the bench for 3 min and then invert 10 more times.
  23. Repeat until most of the beads are resuspended in the elution buffer.
  24. Incubate the tubes at room temperature for 10 min.
  25. Briefly spin down the tubes in a mini centrifuge.
  26. Return the tubes to the magnetic rack.
  27. Allow the beads to separate from the liquid and collect on the side of the tubes for at least 5 min.
  28. Carefully pipette up the supernatant, transfer to new PCR tubes, and place on ice.
  29. Discard the beads.
  30. Add 1 μL of sample to 4 μL of elution buffer to dilute the DNA.
  31. Use 1 μL of the diluted DNA to measure the concentration using the Qubit^TM^ 1× dsDNA HS Assay kit and associated protocol [16].
- **Ligate adapters**
  1. Add 1 μL of annealed barcoded adapter to each sample for a total volume of 31 μL.
*Note: Use different barcodes for samples that will be pooled together.*
  2. Add the following reagents, in order, to a LoBind microcentrifuge tube to make a master mix. Add 1 volume of each reagent per sample with 10% overage:
    T4 DNA ligase reaction buffer 5 μL
    Nuclease-free water 12.5 μL
  3. Add 17.5 μL of master mix to each sample for a total volume of 48.5 μL.
  4. Invert the tube 20 times to mix.
  5. Add 1.5 μL of T4 DNA ligase to each sample for a total volume of 50 μL.
  6. Invert the tube 20 times to mix.
*Note: Do not vortex or flick the tube to avoid shearing the DNA.*
  7. Briefly spin down the tubes in a mini centrifuge.
  8. Place in the thermal cycler and run the following protocol:
    16 °C for 2 h (turn heated lid off for this step)
    65 °C for 10 min
    4 °C hold overnight
- **Pool samples**
  1. Transfer samples to new LoBind microcentrifuge tubes.
  2. In a microcentrifuge, centrifuge the tubes at 14,000× *g* for 5 min.
*Note: Pay attention to the orientation of the tubes.*
  3. Carefully transfer the supernatant to a new LoBind microcentrifuge tube on ice by pipetting up the liquid from the inner-facing tube wall.
*Notes:*
    - *Do not disturb the pellet on the outer-facing wall.*
    - *If multiplexing, pool samples in equimolar amounts at this step. A minimum of 5 μg and a maximum of 25 μg of DNA is recommended per pool/sequencing run.*
  4. Discard the tubes with the pellets.
- **Purify SMRTbell library**
  1. Add elution buffer to the pooled samples for a total volume of 500 μL.
*Note: Additional elution buffer is not necessary if the sample volume is ≥500 μL.*
  2. Mix the AMPure PB beads until a homogeneous solution forms.
  3. Add 0.45× volume of AMPure PB beads to the tube for a total volume of 725 μL.
*Note: The AMPure PB beads will be viscous; pipette slowly to ensure precise volume.*
  4. Vortex or flick the tube to mix the solution.
  5. Briefly spin down the tube in a mini centrifuge.
  6. Vortex the tube at 2,000 rpm for 10 min to bind the DNA to the beads.
*Note: The solution should be homogenous.*
  7. Briefly spin down the tube in a mini centrifuge.
  8. Place the tube into the magnetic rack.
  9. Allow the beads to separate from the liquid and collect on the side of the tube. Wait until the solution appears clear (at least 5 min).
  10. While the tube is in the magnetic rack, slowly remove the supernatant without disturbing the bead pellet and transfer to a new LoBind microcentrifuge tube.
  11. Wash the beads with 1 mL of 80% ethanol by adding to the side opposite of the beads.
*Note: Leave the tube on the magnetic rack during the ethanol washes. Do not invert the tube.*
  12. Wait for 30 s, slowly pipette up the 80% ethanol, and discard.
  13. Repeat the ethanol wash two more times.
  14. Remove the tube from the magnetic rack and briefly spin down the tube in a mini centrifuge.
*Note: Both the beads and ethanol should be at the bottom of the tube.*
  15. Return the tube to the magnetic rack.
  16. With a P20 pipette, slowly pipette up any remaining ethanol and discard.
  17. Repeat steps K14–16 until no ethanol droplets remain.
  18. Remove the tube from the magnetic rack and add 100 μL of elution buffer per 5 μg of input genomic DNA to the beads.
  19. Pipette-mix until the solution is homogeneous.
  20. Incubate at room temperature for 5 min.
  21. Vortex the tube at 2,000 rpm for 1 min.
  22. Briefly spin down the tubes in a mini centrifuge.
  23. Place the tube back on the magnetic rack.
  24. Allow the beads to separate from the liquid and collect on the side of the tubes for at least 5 min.
  25. Carefully pipette up the supernatant, transfer to a new LoBind microcentrifuge tube, and place on ice.
**Safe pausing point:** Store SMRTbell library at 4 °C overnight or at -20 °C for longer storage.
  26. Discard the beads.
- **Nuclease treatment**
  1. Add the following reagents, in order, to the tube with the SMRTbell library. Add 1 volume of each reagent per 100 μL of purified DNA:
    Nuclease-free water 67.2
    CutSmart buffer 20 μL
    Exonuclease III 4.8 μL
    Enzyme A (Enzyme Cleanup kit) 4 μL
    Enzyme B (Enzyme Cleanup kit) 1 μL
    Enzyme C (Enzyme Cleanup kit) 1 μL
    Enzyme D (Enzyme Cleanup kit) 2 μL
  2. Invert the tube 20 times to mix.
  3. Briefly spin down the tubes in a mini centrifuge.
  4. Incubate at 37 °C for 2 h and then place the digested SMRTbell library on ice.
  5. Add 9 μL of SOLu-Trypsin per 200 μL of digested SMRTbell library to the tube.
  6. Invert the tube 20 times to mix.
  7. Briefly spin down the tube in a mini centrifuge.
  8. Incubate at 37 °C for 20 min and place the SMRTbell library on ice.
- **Purify the nuclease-treated SMRTbell library**
  1. Transfer the SMRTbell library to a new LoBind microcentrifuge tube.
  2. Add elution buffer to the sample for a total volume of 500 μL.
*Note: Additional elution buffer is not necessary if the sample volume is ≥500 μL.*
  3. Mix the AMPure PB beads until a homogeneous solution forms.
  4. Add 0.45× volume of AMPure PB beads to the tube for a total volume of 725 μL.
*Note: The AMPure PB beads will be viscous; pipette slowly to ensure precise volume.*
  5. Vortex or flick the tube to mix the solution.
  6. Briefly spin down the tubes in a mini centrifuge.
  7. Vortex the tube at 2,000 rpm for 10 min to bind the DNA to the beads.
*Note: The solution should be homogenous.*
  8. Briefly spin down the tubes in a mini centrifuge.
  9. Place the tube into the magnetic tube rack.
  10. Allow the beads to separate from the liquid and collect on the side of the tube. Wait until the solution appears clear (at least 5 min).
  11. While the tube is in the magnetic rack, slowly remove the supernatant without disturbing the bead pellet and transfer to a new LoBind microcentrifuge tube.
*Note: Save supernatant to repeat purification steps in case insufficient DNA is recovered at the end of the purification protocol.*
  12. Wash the beads with 1 mL of 80% ethanol by adding to the side opposite of the beads.
*Note: Leave the tube on the magnetic rack during the ethanol washes. Do not invert the tube.*
  13. Wait for 30 s, slowly pipette up the 80% ethanol, and discard.
  14. Repeat the ethanol wash two more times.
  15. Remove the tube from the magnetic rack and briefly spin down the tube in a mini centrifuge.
*Note: Both the beads and ethanol should be at the bottom of the tube.*
  16. Return the tube to the magnetic rack.
  17. With a P20 pipette, slowly pipette up any remaining ethanol and discard.
  18. Repeat steps M14–16 until no ethanol droplets remain.
  19. Add 200 μL of elution buffer to the beads.
  20. Pipette-mix until the solution is homogeneous.
  21. Incubate at room temperature for 5 min.
  22. Vortex the tube at 2,000 rpm for 1 min.
  23. Briefly spin down the tubes in a mini centrifuge.
  24. Place the tube back on the magnetic rack.
  25. Allow the beads to separate from the liquid and collect on the side of the tubes for at least 5 min.
  26. Carefully pipette up the supernatant, transfer to a new LoBind microcentrifuge tube, and place on ice.
  27. Discard the beads.
- **Purify the nuclease-treated SMRTbell library a second time**
  1. Mix the AMPure PB beads until a homogeneous solution forms.
  2. Add 0.42× of AMPure PB beads to the tube from the first round of purification for a total volume of 284 μL.
*Note: The AMPure PB beads will be viscous; pipette slowly to ensure precise volume.*
  3. Vortex or flick the tube to mix the solution.
  4. Briefly spin down the tube in a mini centrifuge.
  5. Vortex the tube at 2,000 rpm for 10 min to bind the DNA to the beads.
*Note: The solution should be homogenous.*
  6. Briefly spin down the tube in a mini centrifuge.
  7. Place the tube into the magnetic rack.
  8. Allow the beads to separate from the liquid and collect on the side of the tube. Wait until the solution appears clear (at least 5 min).
  9. While the tube is in the magnetic rack, slowly remove the supernatant without disturbing the bead pellet and transfer to a new LoBind microcentrifuge tube.
  10. Wash the beads with 1 mL of 80% ethanol by adding to the side opposite of the beads.
*Note: Leave the tube on the magnetic rack during the ethanol washes. Do not invert the tube.*
  11. Wait for 30 s, slowly pipette up the 80% ethanol, and discard.
  12. Repeat the ethanol wash two more times.
  13. Remove the tube from the magnetic rack and briefly spin down the tube in a mini centrifuge.
*Note: Both the beads and ethanol should be at the bottom of the tube.*
  14. Return the tube to the magnetic rack.
  15. With a P20 pipette, slowly pipette up any remaining ethanol and discard.
  16. Repeat steps N13–15 until no ethanol droplets remain.
  17. Add 6.3 μL of elution buffer to the beads.
  18. Pipette-mix until the solution is homogeneous.
  19. Incubate at room temperature for 5 min.
  20. Vortex the tube at 2,000 rpm for 1 min.
  21. Briefly spin down the tube in a mini centrifuge.
  22. Place the tube back on the magnetic rack.
  23. Allow the beads to separate from the liquid and collect on the side of the tubes for at least 5 min.
  24. Carefully pipette up the supernatant, transfer to a new LoBind microcentrifuge tube, and place on ice.
**Safe pausing point:** Store SMRTbell library at 4 °C overnight or at -20 °C for longer storage.
    If sending samples to a sequencing core, ship on dry ice for next-day delivery during this safe pausing point. The sequencing core should complete the rest of the protocol prior to sequencing.
  25. Discard the beads.
- **Primer annealing**
  1. Add 1 μL of sequencing primer v4 stock to 29 μL of elution buffer.
  2. Add the following reagents, in order, to a PCR tube:
    10× primer buffer v2 36 μL
    Diluted sequencing primer v4 18 μL
  3. Pipette or flick the tube to mix the solution.
  4. Briefly spin down the tubes in a mini centrifuge.
  5. Place in the thermal cycler and run the following protocol:
    80 °C for 2 min
    4 °C hold
  6. Transfer to a new LoBind microcentrifuge tube and place on ice.
*Note: Store remaining conditioned sequencing primer at -20 °C for a maximum of 30 days.*
  7. Add the following reagents, in order, to a new PCR tube for a total volume of 9 μL:
    Conditioned sequencing primer v4 (from step O6) 2.7 μL
    SMRTbell library (from section L) 6.3 μL
  8. Pipette or flick the tube to mix the solution.
  9. Briefly spin down the tubes in a mini centrifuge.
  10. Place in the thermal cycler and run the following protocol:
    20 °C for 1 h
    4 °C hold
- **Polymerase binding**
  **For Sequel**
  1. Add 1 μL of Sequel DNA Polymerase 3.0 to 29 μL of Sequel Binding Buffer and place on ice.
*Note: Diluted Sequel DNA Polymerase 3.0 must be used immediately; discard any excess.*
  2. Add the following reagents, in order, to the PCR tube with the primer-annealed SMRTbell library for a total volume of 13.5 μL:
    Sequel Binding Buffer 1.5 μL
    DTT 1.5 μL
    Sequel dNTP 1.5 μL
  3. Pipette or flick the tube to mix the solution.
  4. Add 1.5 μL of Diluted Sequel DNA Polymerase 3.0 for a total volume of 15 μL.
  5. Pipette or flick the tube to mix the solution.
  6. Briefly spin down the tubes in a mini centrifuge.
  7. Place the sample complex in the thermal cycler and run the following protocol:
    30 °C for 4 h
    4 °C hold (or hold on ice) until ready for the purification step
**For Sequel II and IIe**
  1. Add 1 μL of Sequel II DNA Polymerase 2.0 to 29 μL of Sequel Binding Buffer and place on ice.
*Note: Diluted Sequel II DNA Polymerase 2.0 must be used immediately; discard excess.*
  2. Add the following reagents, in order, to the PCR tube with the primer-annealed SMRTbell library for a total volume of 13.5 μL:
    Sequel Binding Buffer 1.5 μL
    DTT 1.5 μL
    Sequel dNTP 1.5 μL
  3. Pipette or flick the tube to mix the solution.
  4. Add 1.5 μL of diluted Sequel II DNA Polymerase 2.0 for a total volume of 15 μL.
  5. Pipette or flick the tube to mix the solution.
  6. Briefly spin down the tubes in a mini centrifuge.
  7. Place the sample complex in the thermal cycler and run the following protocol:
    30 °C for 4 h
    4 °C hold (or hold on ice) until ready for the purification step
- **Purify of SMRTbell complex**
  **For Sequel**
  1. Add 35 μL of Sequel Complex Dilution Buffer to a new LoBind microcentrifuge tube.
  2. Add the total volume (15 μL) of the sample complex to the tube for a total volume of 50 μL.
  3. Mix the AMPure PB beads until a homogeneous solution forms.
  4. Add 0.6× of AMPure PB beads to the diluted sample complex for a total volume of 80 μL.
*Note: The AMPure PB beads will be viscous; pipette slowly to ensure precise volume.*
  5. Pipette or flick the tube to mix the solution.
  6. Incubate the tube at room temperature for 5 min.
  7. Place the tube into the magnetic rack.
  8. Allow the beads to separate from the liquid and collect on the side of the tube. Wait until the solution appears clear (at least 2 min).
  9. While the tube is in the magnetic rack, slowly remove the supernatant without disturbing the bead pellet and discard.
  10. Briefly spin down the tube in a mini centrifuge.
  11. Place the tube back into the magnetic rack.
  12. Remove any remaining supernatant and discard.
  13. Resuspend the beads in 81 μL of Sequel Complex Dilution Buffer immediately.
  14. Pipette or flick the tube to mix the solution.
  15. Incubate at room temperature for 15 min.
  16. Place the tube into the magnetic rack.
  17. Allow the beads to separate from the liquid and collect on the side of the tube. Wait until the solution appears clear (at least 1 min).
  18. Carefully pipette up the supernatant, transfer to a new LoBind microcentrifuge tube, and place on ice.
  19. On ice, dilute the Sequel DNA Internal Control Complex 3.0 10,000-fold by performing two 100-fold serial dilutions in Sequel Complex Dilution Buffer.
  20. Add 3 μL of the diluted DNA internal control complex and 1 μL of carrier DNA to the tube with the eluted sample for a total volume of 85 μL.
  21. Transfer the sample to a sample plate.
  22. Cover the plate and store at 4 °C or on ice until sequencing.
  **For Sequel II and IIe**
  1. Add 35 μL Sequel Complex Dilution Buffer to a new LoBind microcentrifuge tube.
  2. Add the total volume (15 μL) of the sample complex to the tube for a total volume of 50 μL.
  3. Mix the AMPure PB beads until a homogeneous solution forms.
  4. Add 0.6× of AMPure PB beads to the diluted sample complex for a total volume of 80 μL.
*Note: The AMPure PB beads will be viscous; pipette slowly to ensure precise volume.*
  5. Pipette or flick the tube to mix the solution.
  6. Incubate the tube at room temperature for 5 min.
  7. Place the tube into the magnetic rack.
  8. Allow the beads to separate from the liquid and collect on the side of the tube. Wait until the solution appears clear (at least 2 min).
  9. While the tube is in the magnetic rack, slowly remove the supernatant without disturbing the bead pellet and discard.
  10. Briefly spin down the tube in a mini centrifuge.
  11. Place the tube back into the magnetic rack.
  12. Remove any remaining supernatant and discard.
  13. Resuspend the beads in 109.6 μL of Sequel Complex Dilution Buffer immediately.
  14. Pipette or flick the tube to mix the solution.
  15. Incubate at room temperature for 15 min.
  16. Place the tube back into the magnetic rack.
  17. Allow the beads to separate from the liquid and collect on the side of the tube. Wait until the solution appears clear (at least 1 min).
  18. Carefully pipette up the supernatant, transfer to a new LoBind microcentrifuge tube, and place on ice.
  19. On ice, dilute the Sequel II DNA Internal Control Complex 1.0 10,000-fold by performing two 100-fold serial dilutions in Sequel Complex Dilution Buffer.
  20. Add 4 μL of the diluted DNA internal control complex and 1.4 μL of carrier DNA to the tube with the eluted sample for a total volume of 115 μL.
  21. Transfer the sample to a sample plate.
  22. Cover the plate and keep at 4 °C or on ice until sequencing.
- **Sequencing**
  **For Sequel**
  1. Sequence plate on the Sequel system with the following settings:
    - SMRT Link Run Design > Advanced Options > Immobilization Time > 4 h.
    - No pre-extension.
    - Movie collection time: 20 h.
    - Generate HiFi reads > IN SMRT LINK.
  **For Sequel II**
  1. Sequence on the Sequel II system with the following settings:
    - SMRT Link Run Design > Advanced Options > Immobilization Time > 4 h.
    - No pre-extension.
    - Movie collection time: 20 h.
    - Generate HiFi reads > IN SMRT LINK.
  **For Sequel IIe**
  1. Sequence on the Sequel IIe system with the following settings:
    - SMRT Link Run Design > Advanced Options > Immobilization Time > 4 h.
    - No pre-extension.
    - Movie collection time: 20 h.
    - Generate HiFi reads > ON INSTRUMENT.

## Data analysis

Sequencing data is processed using SMRTLink according to the SMRTLink User Guide [18] and as described here. High-quality single-molecule circular consensus sequences (CCS or HiFi reads) are generated from the raw data employing ccs. To ensure the integrity of the data, only sequences achieving a minimum of three passes and a mean read accuracy of QV20 are retained.

**For Sequel and Sequel II**

Process raw subreads with the following steps and settings:

SMRT Link Analysis > Data Utility > Circular Consensus Sequencing > Advanced Options > MinPasses > 3.

Min QV: 20.

**For Sequel IIe:** CCS are generated on instrument.

Subsequently, the reads are demultiplexed using lima and aligned to the reference genome with pbmm2.

**For Sequel and Sequel II**

1. Demultiplex samples with the following settings:
  SMRT Link Analyses > Data Utility > Demultiplex > same on both sides.
2. Align HiFi reads for each sample to the reference with the following settings:
  SMRT Link Analysis > One Analysis Per Data Set – Identical Parameters > HiFi Mapping > Reference > GrCH38_no_alts.

**For Sequel IIe**

1. Demultiplex samples with the following settings:
  SMRT Link Analyses > Data Utility > Demultiplex > same on both sides.
2. Align HiFi reads for each sample to the reference with the following settings:
  SMRT Link Analysis > One Analysis Per Data Set – Identical Parameters > HiFi Mapping > Reference > GrCH38_no_alts.
  Single-molecule sequencing reads can be assembled into waterfall plots for visualization of the target region (Figure 2).
  Python extractRegion.py [mapped sample] [ref] [coords] > python waterfall.py [motifs].
  **Figure 2.**
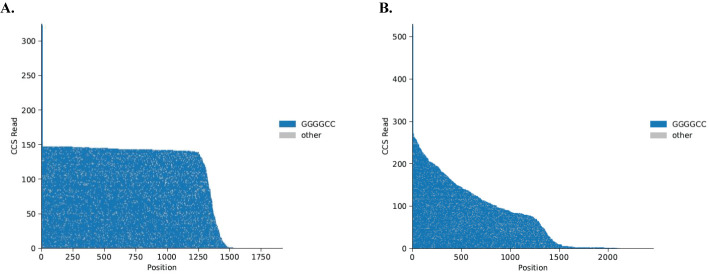
Example of PacBio Sequel II sequencing output. Waterfall plots of single-molecule sequencing reads. Each horizontal line represents one molecule sequenced, anchored at a non-repeat region 5′ to the *C9orf72* repeat expansion. The X-axis shows the nucleotide position compared to the anchor. The Y-axis shows the CCS read count. The grey color represents changes in the sequence other than GGGGCC repeat, which are likely to be a sequencing error. **(A)** NoAmp sequencing of patient iPSC DNA with two repeats on the normal allele (tall blue tail reaching 300 CCS reads) and approximately 250 repeats on the expanded allele. **(B)** NoAmp sequencing of patient iPSC DNA showing repeat lengths from 2 to 250 representing mosaicism in this line.

The program primrose can be used to predict 5-methylcytosine signatures in HiFi reads using a convolutional neural network applied to multi-pass kinetics data for each read. Methylation data can be further characterized using pb-CpG-tools, which rely on tags generated by primrose for calculating the probability of 5mC methylation. Supplemental Figure 12 of Sachdev et al. [10] shows an example of methylation data obtained using this protocol.

## Validation of protocol

This protocol has been used and validated in the following research articles:

- Salomonsson et al. [5]. Validated assays for the quantification of *C9orf72* human pathology. *Sci Rep*. 14: 828.
- Sachdev et al. [10]. Reversal of *C9orf72* mutation-induced transcriptional dysregulation and pathology in cultured human neurons by allele-specific excision. *Proc Natl Acad Sci USA.* 121(17): e2307814121

## General notes and troubleshooting

**General notes**

1. Room temperature refers to 20–22 °C.
2. We have validated multiplexes up to 10 samples but recommend multiplexing a maximum of 5 samples for optimal sequencing coverage.
3. Before using genomic DNA, allow samples to reach room temperature and invert the tube 20 times to mix.
4. Use a benchtop cooler to keep all enzymes at -20 °C during the protocol.
5. Fresh 80% ethanol solutions (stored for up to three days) must be used for all wash steps.
6. For all enzymatic reactions, use a thermocycler or heat block with a heated lid set to 10 °C above the incubation temperature whenever possible.
7. sgRNAs and reagents used in sections E, F, and G should be kept on ice at all times.
8. Let AMPure PB beads and elution buffer reach room temperature prior to use.
9. The purification steps are necessary to eliminate any unwanted DNA fragments and failed ligation products. The second purification uses a smaller ratio of beads to sample volume (0.42× instead of 0.45×) than the first step, further selecting for larger fragments to enrich on-target reads.
10. Do not over-dry the AMPure PB beads during purification.
11. Unless there is a safe pausing point noted, immediately proceed to the next step of the protocol.

**Troubleshooting**

1. If genomic DNA samples show smearing (indicating DNA shearing) when run on a 1% gel, collect new samples. Avoiding the vortexing of genomic DNA samples and freeze-thaw cycles can prevent smearing.
2. If genomic DNA samples have fragment sizes <50 kb, the starting input amount can be increased to generate more reads. If fragments are significantly degraded (fragment sizes <20 kb), collect new samples. Avoiding the vortexing of genomic DNA samples and freeze-thaw cycles can prevent short fragment sizes.

## References

[1] DeJesus-Hernandez M., Mackenzie I. R., Boeve B. F., Boxer A. L., Baker M., Rutherford N. J., Nicholson A. M., Finch N. A., Flynn H., Adamson J., .(2011). Expanded GGGGCC Hexanucleotide Repeat in Noncoding Region of *C9orf72* Causes Chromosome 9p-Linked FTD and ALS. Neuron. 72(2): 245-256. 21944778 10.1016/j.neuron.2011.09.011 PMC3202986

[2] Renton A. E., Majounie E., Waite A., Simón-Sánchez J., Rollinson S., Gibbs J. R., Schymick J. C., Laaksovirta H., van Swieten J. C., Myllykangas L., .(2011). A Hexanucleotide Repeat Expansion in *C9orf72* Is the Cause of Chromosome 9p21-Linked ALS-FTD. Neuron. 72(2): 257-268. 21944779 10.1016/j.neuron.2011.09.010 PMC3200438

[3] Majounie E., Renton A. E., Mok K., Dopper E. G., Waite A., Rollinson S., Chiò A., Restagno G., Nicolaou N., Simon-Sanchez J., .(2012). Frequency of the *C9orf72* hexanucleotide repeat expansion in patients with amyotrophic lateral sclerosis and frontotemporal dementia: a cross-sectional study. Lancet Neurol. 11(4): 323-330. 22406228 10.1016/S1474-4422(12)70043-1 PMC3322422

[4] Iacoangeli A., Al Khleifat A., Jones A. R., Sproviero W., Shatunov A., Opie-Martin S., Morrison K. E., Shaw P. J., Shaw C. E., .(2019). *C9orf72* intermediate expansions of 24–30 repeats are associated with ALS. Acta Neuropathol Commun. 7(1): 115. 31315673 10.1186/s40478-019-0724-4 PMC6637621

[5] Salomonsson S. E., Maltos A. M., Gill K., Aladesuyi Arogundade O., Brown K. A., Sachdev A., Sckaff M., Lam K. J. K., Fisher I. J., Chouhan R. S., .(2024). Validated assays for the quantification of *C9orf72* human pathology. Sci Rep. 14(1): 828. 38191789 10.1038/s41598-023-50667-3 PMC10774390

[6] Bram E., Javanmardi K., Nicholson K., Culp K., Thibert J. R., Kemppainen J., Le V., Schlageter A., Hadd A., Latham G. J., .(2018). Comprehensive genotyping of the *C9orf72* hexanucleotide repeat region in 2095 ALS samples from the NINDS collection using a two-mode, long-read PCR assay. Amyotroph Lateral Scler Frontotemporal Degener. 20: 107-114. 30430876 10.1080/21678421.2018.1522353 PMC6513680

[7] Buchman V. L., Cooper-Knock J., Connor-Robson N., Higginbottom A., Kirby J., Razinskaya O. D., Ninkina N. and Shaw P. J. (2013). Simultaneous and independent detection of *C9orf72* alleles with low and high number of GGGGCC repeats using an optimised protocol of Southern blot hybridisation. Mol Neurodegener. 8(1): 12. 23566336 10.1186/1750-1326-8-12 PMC3626718

[8] Ebbert M. T. W., Farrugia S. L., Sens J. P., Jansen-West K., Gendron T. F., Prudencio M., McLaughlin I. J., Bowman B., Seetin M., DeJesus-Hernandez M., .(2018). Long-read sequencing across the *C9orf72*‘GGGGCC’ repeat expansion: implications for clinical use and genetic discovery efforts in human disease. Mol Neurodegener. 13(1): 46. 30126445 10.1186/s13024-018-0274-4 PMC6102925

[9] DeJesus-Hernandez M., Aleff R. A., Jackson J. L., Finch N. A., Baker M. C., Gendron T. F., Murray M. E., McLaughlin I. J., Harting J. R., Graff-Radford N. R., .(2021). Long-read targeted sequencing uncovers clinicopathological associations for *C9orf72*-linked diseases. Brain 144(4): 1082-1088. 33889947 10.1093/brain/awab006 PMC8105038

[10] Sachdev A., Gill K., Sckaff M., Birk A. M., Aladesuyi Arogundade O., Brown K. A., Chouhan R. S., Issagholian-Lewin P. O., Patel E., Watry H. L., .(2024). Reversal of *C9orf72* mutation-induced transcriptional dysregulation and pathology in cultured human neurons by allele-specific excision. Proc Natl Acad Sci USA. 121(17): e2307814121. 38621131 10.1073/pnas.2307814121 PMC11047104

[11] Hafford-Tear N. J., Tsai Y. C., Sadan A. N., Sanchez-Pintado B., Zarouchlioti C., Maher G. J., Liskova P., Tuft S. J., Hardcastle A. J., Clark T. A., .(2019). CRISPR/Cas9-targeted enrichment and long-read sequencing of the Fuchs endothelial corneal dystrophy–associated *TCF4* triplet repeat. Genet Med. 21(9): 2092-2102. 30733599 10.1038/s41436-019-0453-x PMC6752322

[12] Tsai Y. C., de Pontual L., Heiner C., Stojkovic T., Furling D., Bassez G., Gourdon G. and Tomé S. (2022). Identification of a CCG-Enriched Expanded Allele in Patients with Myotonic Dystrophy Type 1 Using Amplification-Free Long-Read Sequencing. J Mol Diagn. 24(11): 1143-1154. 36084803 10.1016/j.jmoldx.2022.08.003

[13] PacBio(2024). Generating PureTarget^TM^ repeat expansion panel libraries.

[14] Fisher I. J., Salomonsson S. and Clelland C. D. (2023). iPSC Cell Culture Protocol. Protocols.io. dx.doi.org/10.17504/protocols.io.36wgqj2n3vk5/v1.

[15] QIAGEN(2015). QIAGEN^®^ Genomic DNA Handbook.

[16] Thermo Fisher Scientific(2020). Qubit^TM^ 1X dsDNA HS Assay Kits User Guide.

[17] Edvotek(2020). Quick Guide: SYBR^®^ Safe DNA Stain.

[18] PacBio(2023). SMRT^®^ Link User Guide v12.0.

